# 8-(Diphenyl­phosphan­yl)quinoline

**DOI:** 10.1107/S1600536810040237

**Published:** 2010-10-20

**Authors:** Samik Nag, Mihaela Cibian, Garry S. Hanan

**Affiliations:** a Department of Chemical Sciences, Sikkim University, 6th Mile, Tadong, Gangtok, Sikkim 737 102, India; b Département de Chimie, Université de Montréal, CP 6128, Succ., Centre-ville, Montréal, Québec, Canada H3C 3J7

## Abstract

The title compound, C_21_H_16_NP, is a known P—N chelator and various crystal structures of its metal complexes have been reported. However, no crystallographic evidence of the free ligand has been given to date. The phenyl rings are almost orthogonal to one another [dihedral angle = 88.9 (1)°], and they are twisted from the mean plane of the quinoline by 80.5 (1) and 76.3 (1)°.

## Related literature

Synthetic details regarding this compound were reported by Issleib & Haftendorn (1970 ▶); Feltham & Metzger (1971 ▶); Lai *et al.* (2001 ▶); Lord *et al.* (2009 ▶). For the crystal structures of some of its metal complexes, see: Hudali *et al.* (1979 ▶); Sun *et al.* (2002 ▶); Suzuki (2004 ▶); Suzuki *et al.* (2009 ▶); Canovese *et al.* (2008 ▶); Qin *et al.* (2009 ▶); Tsukuda *et al.* (2009 ▶). The propeller-type conformation of the title compound is characteristic for tris-(ar­yl)-substituted phosphines, see: Beck *et al.* (2008 ▶). For C—P—C angles in related structures, see: Van Allen & Venkataraman (2003 ▶); Chuit *et al.* (1993 ▶). For hydrogen bonds, see: Desiraju & Steiner (1999 ▶). For a description of the Cambridge Structural Database, see: Allen (2002 ▶).
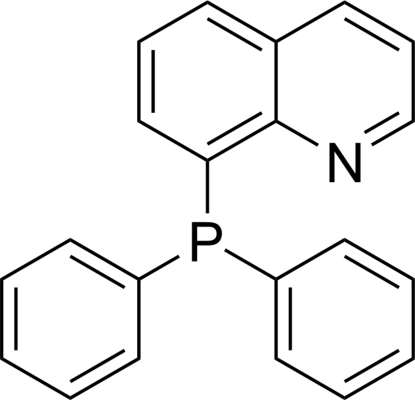


## Experimental

### 

#### Crystal data

C_21_H_16_NP
*M* *_r_* = 313.32
Monoclinic, 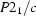

*a* = 10.7804 (2) Å
*b* = 16.6905 (3) Å
*c* = 9.7753 (2) Å
β = 112.651 (1)°
*V* = 1623.21 (5) Å^3^
*Z* = 4
Cu *K*α radiation
μ = 1.47 mm^−1^
*T* = 150 K
0.20 × 0.18 × 0.12 mm

#### Data collection

Bruker APEXII diffractometer
Absorption correction: multi-scan (*SADABS*; Sheldrick, 1996 ▶) *T*_min_ = 0.658, *T*_max_ = 0.839
20905 measured reflections
3170 independent reflections
3067 reflections with *I* > 2σ(*I*)
*R*_int_ = 0.028

#### Refinement

*R*[*F* ^2^ > 2σ(*F* ^2^)] = 0.041
*wR*(*F* ^2^) = 0.106
*S* = 1.07
3170 reflections
209 parameters
H-atom parameters constrained
Δρ_max_ = 0.28 e Å^−3^
Δρ_min_ = −0.34 e Å^−3^

### 

Data collection: *APEX2* (Bruker 2007 ▶); cell refinement: *SAINT* (Bruker, 2007 ▶); data reduction: *SAINT*; program(s) used to solve structure: *SHELXTL* (Sheldrick, 2008 ▶); program(s) used to refine structure: *SHELXTL*; molecular graphics: *SHELXTL*; software used to prepare material for publication: *UdMX* (Maris, 2004 ▶).

## Supplementary Material

Crystal structure: contains datablocks I, global. DOI: 10.1107/S1600536810040237/nk2063sup1.cif

Structure factors: contains datablocks I. DOI: 10.1107/S1600536810040237/nk2063Isup2.hkl

Additional supplementary materials:  crystallographic information; 3D view; checkCIF report

## Figures and Tables

**Fig. 1.**
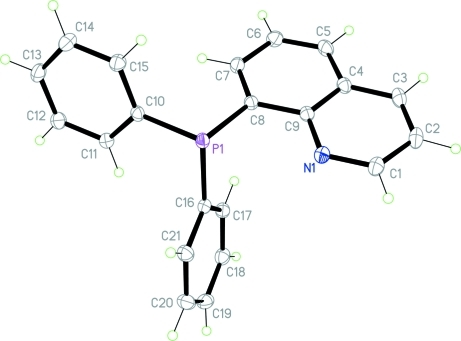
The molecular structure of the title compound (30% probability displacement ellipsoids).

## supplementary crystallographic information

### Comment

Bearing both imine and phosphine moieties, 8-quinolylphosphine derivatives are good chelators for transition metals. (Hudali *et al.*, 1979) Their complexes have important photochemical and photophysical properties and are widely used in chemical industry (catalysis, functional materials, *etc*). (Canovese *et al.*, 2008; Qin *et al.*, 2009; Tsukuda *et al.*, 2009). For the specific example of the 8-(diphenylphosphino)quinoline, although crystallographic evidence of various of its metal complexes exists (Suzuki, 2004; Suzuki *et al.*, 2009, Canovese *et al.*, 2008), this is the first report of the free ligand structure (Figure 1).

The structure has a propeller-type conformation, characteristic for tris-(aryl) substituted phosphines (Beck *et al.*, 2008). The P—C bond lengths are within normal ranges for similar arylphosphines (1.81–1.87 Å) (CSD search 09/2010, 29 compounds; Allen, 2002). The phosphorus presents a pyramidal configuration, with the average value of C—P—C angles of 101.7°, in comparison to 100.7° calculated for naphtalene-1yl(diphenylphosphane) (Van Allen & Venkataraman, 2003) and 103.4° for triphenylphosphine (Chuit *et al.*,1993).

It is worth mentioning the almost orthogonal position of the phenyl rings to one another (88.9 (1)°), and their tilt with respect to the mean plane of the quinoline by 80.5 (1)° and 76.3 (1)°, maximizing the intramolecular CH/π interactions. The structure is also stabilized by intermolecular CH/π interactions between the proton H3 of the quinolyl ring and the π system of an adjacent phenyl (H3···*Cg* 2.8 Å, C3—H3···*Cg* 170 (1)°). It is also to be noted the short contact of 2.9 Å, at the limit of the van der Waals radius (3.0 Å), between the phosphorus atom and the quinolyl hydrogen H2 of an adjacent molecule. The distance P···H2 of 2.9 Å and the angle C2—H2···P of 161 (1)° could place this contact it the category of weak donor – weak acceptor interactions. (Desiraju & Steiner, 1999)

### Experimental

The title compound, 8-(diphenylphosphino)quinoline, was synthesized by the reaction of 8-(trifluoromethylsulfonyl)quinoline with tetrakis-triphenylphosphine palladium in presence of zinc cyanide, as a byproduct of 8-cyanoquinoline (Lord *et al.* 2009). 8-(Trifluoromethylsulfonyl)quinoline (2.0 g, 7.3 mmol), zinc cyanide (0.54 g, 4.6 mmol) and tetrakis-triphenylphosphine palladium (0.84 g, 0.73 mmol) were taken in dry DMF (15 ml) and refluxed under nitrogen for 2 h. The reaction mixture was cooled and poured into water (150 ml). Aqueous H_2_SO_4_ (2*M*) (15 ml) was added and the mixture was stirred for 5 min. This was then extracted with EtOAc (2 *x* 100 ml), washed with brine and dried over anhydrous MgSO_4_. Evaporation of the solvent gave a brown gummy solid. This was subjected to column chromatography on SiO_2_ with 30% EtOAc in n-hexane as eluent. The first band contained the title compound. Solvent evaporation at room temperature gave off-white X-ray quality crystals.

### Refinement

The H atoms were positioned geometrically (C—H 0.95 Å) and included in the refinement in the riding model approximation; their temperature displacement parameters were set to 1.2 times the equivalent isotropic temperature factors of the parent site.

### Crystal data

**Table:**

C_21_H_16_NP	*F*(000) = 656
*M**_r_* = 313.32	*D*_x_ = 1.282 Mg m^−^^3^
Monoclinic, *P*2_1_/*c*	Cu *K*α radiation, λ = 1.54178 Å
Hall symbol: -P 2ybc	Cell parameters from 9934 reflections
*a* = 10.7804 (2) Å	θ = 4.4–72.3°
*b* = 16.6905 (3) Å	µ = 1.47 mm^−^^1^
*c* = 9.7753 (2) Å	*T* = 150 K
β = 112.651 (1)°	Block, colourless
*V* = 1623.21 (5) Å^3^	0.20 × 0.18 × 0.12 mm
*Z* = 4	

### Data collection

**Table:**

Bruker APEXII diffractometer	3170 independent reflections
Radiation source: Rotating Anode	3067 reflections with *I* > 2σ(*I*)
Helios optics	*R*_int_ = 0.028
Detector resolution: 5.5 pixels mm^-1^	θ_max_ = 72.4°, θ_min_ = 4.4°
ω scans	*h* = −13→13
Absorption correction: multi-scan (*SADABS*; Sheldrick, 1996)	*k* = −20→20
*T*_min_ = 0.658, *T*_max_ = 0.839	*l* = −12→12
20905 measured reflections	

### Refinement

**Table:**

Refinement on *F*^2^	Secondary atom site location: difference Fourier map
Least-squares matrix: full	Hydrogen site location: inferred from neighbouring sites
*R*[*F*^2^ > 2σ(*F*^2^)] = 0.041	H-atom parameters constrained
*wR*(*F*^2^) = 0.106	*w* = 1/[σ^2^(*F*_o_^2^) + (0.0691*P*)^2^ + 0.4334*P*] where *P* = (*F*_o_^2^ + 2*F*_c_^2^)/3
*S* = 1.07	(Δ/σ)_max_ = 0.001
3170 reflections	Δρ_max_ = 0.28 e Å^−^^3^
209 parameters	Δρ_min_ = −0.34 e Å^−^^3^
0 restraints	Extinction correction: *SHELXTL* (Sheldrick, 2008), Fc^*^=kFc[1+0.001xFc^2^λ^3^/sin(2θ)]^-1/4^
Primary atom site location: structure-invariant direct methods	Extinction coefficient: 0.0148 (8)

### Special details

**Table:**

Experimental. X-ray crystallographic data for I were collected from a single-crystal sample, which was mounted on a loop fiber. Data were collected using a Bruker Platform diffractometer, equipped with a Bruker *SMART* 4 K Charged-Coupled Device (CCD) Area Detector using the program *APEX2* and a Nonius FR591 rotating anode equiped with a Montel 200 optics The crystal-to-detector distance was 5.0 cm, and the data collection was carried out in 512 *x* 512 pixel mode. The initial unit-cell parameters were determined by a least-squares fit of the angular setting of strong reflections, collected by a 10.0 degree scan in 33 frames over four different parts of the reciprocal space (132 frames total). One complete sphere of data was collected, to better than 0.80Å resolution.
Geometry. All e.s.d.'s (except the e.s.d. in the dihedral angle between two l.s. planes) are estimated using the full covariance matrix. The cell e.s.d.'s are taken into account individually in the estimation of e.s.d.'s in distances, angles and torsion angles; correlations between e.s.d.'s in cell parameters are only used when they are defined by crystal symmetry. An approximate (isotropic) treatment of cell e.s.d.'s is used for estimating e.s.d.'s involving l.s. planes.
Refinement. Refinement of *F*^2^ against ALL reflections. The weighted *R*-factor *wR* and goodness of fit *S* are based on *F*^2^, conventional *R*-factors *R* are based on *F*, with *F* set to zero for negative *F*^2^. The threshold expression of *F*^2^ > σ(*F*^2^) is used only for calculating *R*-factors(gt) *etc*. and is not relevant to the choice of reflections for refinement. *R*-factors based on *F*^2^ are statistically about twice as large as those based on *F*, and *R*- factors based on ALL data will be even larger.

### Fractional atomic coordinates and isotropic or equivalent isotropic displacement parameters (Å^2^)

**Table:**

	*x*	*y*	*z*	*U*_iso_*/*U*_eq_	
P1	0.85952 (3)	1.005358 (18)	0.64679 (3)	0.02256 (14)	
N1	0.93166 (11)	0.84034 (6)	0.73402 (12)	0.0271 (3)	
C1	0.97809 (14)	0.76959 (8)	0.79118 (16)	0.0314 (3)	
H1	1.0426	0.7678	0.8901	0.038*	
C2	0.93792 (15)	0.69649 (8)	0.71462 (17)	0.0352 (3)	
H2	0.9740	0.6472	0.7619	0.042*	
C3	0.84690 (14)	0.69733 (8)	0.57230 (17)	0.0337 (3)	
H3	0.8207	0.6488	0.5181	0.040*	
C4	0.79150 (13)	0.77131 (7)	0.50553 (15)	0.0270 (3)	
C5	0.69380 (14)	0.77770 (8)	0.35912 (16)	0.0319 (3)	
H5	0.6625	0.7309	0.3007	0.038*	
C6	0.64444 (14)	0.85111 (8)	0.30167 (15)	0.0320 (3)	
H6	0.5788	0.8549	0.2035	0.038*	
C7	0.69018 (13)	0.92151 (8)	0.38711 (14)	0.0276 (3)	
H7	0.6544	0.9720	0.3455	0.033*	
C8	0.78570 (12)	0.91807 (7)	0.52963 (13)	0.0229 (3)	
C9	0.83787 (12)	0.84184 (7)	0.59135 (14)	0.0238 (3)	
C10	0.76718 (12)	1.08881 (7)	0.52806 (13)	0.0236 (3)	
C11	0.66326 (13)	1.13098 (8)	0.54748 (14)	0.0282 (3)	
H11	0.6300	1.1134	0.6193	0.034*	
C12	0.60791 (15)	1.19864 (8)	0.46244 (16)	0.0337 (3)	
H12	0.5381	1.2273	0.4775	0.040*	
C13	0.65439 (15)	1.22434 (9)	0.35592 (16)	0.0343 (3)	
H13	0.6172	1.2708	0.2988	0.041*	
C14	0.75532 (14)	1.18199 (9)	0.33304 (16)	0.0345 (3)	
H14	0.7853	1.1985	0.2580	0.041*	
C15	0.81267 (13)	1.11546 (8)	0.41960 (15)	0.0298 (3)	
H15	0.8836	1.0877	0.4051	0.036*	
C16	0.77946 (14)	1.00053 (7)	0.78177 (15)	0.0243 (3)	
C17	0.66076 (13)	0.95868 (7)	0.75734 (15)	0.0275 (3)	
H17	0.6172	0.9308	0.6666	0.033*	
C18	0.60580 (14)	0.95742 (8)	0.86437 (16)	0.0326 (3)	
H18	0.5249	0.9288	0.8467	0.039*	
C19	0.66880 (17)	0.99806 (8)	0.99754 (17)	0.0361 (4)	
H19	0.6311	0.9971	1.0708	0.043*	
C20	0.78696 (15)	1.04008 (9)	1.02311 (15)	0.0367 (3)	
H20	0.8300	1.0680	1.1138	0.044*	
C21	0.84206 (14)	1.04117 (8)	0.91598 (15)	0.0308 (3)	
H21	0.9230	1.0698	0.9341	0.037*	

### Atomic displacement parameters (Å^2^)

**Table:**

	*U*^11^	*U*^22^	*U*^33^	*U*^12^	*U*^13^	*U*^23^
P1	0.0238 (2)	0.0191 (2)	0.0238 (2)	−0.00020 (10)	0.00809 (15)	−0.00011 (10)
N1	0.0270 (5)	0.0253 (5)	0.0283 (6)	0.0019 (4)	0.0100 (4)	0.0023 (4)
C1	0.0292 (7)	0.0299 (7)	0.0345 (7)	0.0042 (5)	0.0115 (6)	0.0063 (5)
C2	0.0342 (7)	0.0243 (6)	0.0477 (8)	0.0069 (5)	0.0166 (6)	0.0085 (6)
C3	0.0358 (7)	0.0210 (6)	0.0470 (8)	−0.0002 (5)	0.0190 (6)	−0.0024 (6)
C4	0.0284 (6)	0.0223 (6)	0.0335 (7)	−0.0013 (5)	0.0155 (5)	−0.0014 (5)
C5	0.0360 (7)	0.0257 (6)	0.0338 (7)	−0.0057 (5)	0.0131 (6)	−0.0084 (5)
C6	0.0338 (7)	0.0320 (7)	0.0254 (6)	−0.0043 (5)	0.0062 (5)	−0.0024 (5)
C7	0.0300 (6)	0.0237 (6)	0.0271 (6)	−0.0001 (5)	0.0089 (5)	0.0016 (5)
C8	0.0248 (6)	0.0206 (6)	0.0246 (6)	−0.0006 (4)	0.0109 (5)	−0.0008 (4)
C9	0.0241 (6)	0.0227 (6)	0.0270 (6)	−0.0006 (4)	0.0124 (5)	−0.0003 (5)
C10	0.0256 (6)	0.0189 (6)	0.0244 (6)	−0.0023 (4)	0.0075 (5)	−0.0023 (4)
C11	0.0333 (7)	0.0252 (6)	0.0281 (6)	0.0022 (5)	0.0140 (5)	0.0010 (5)
C12	0.0372 (7)	0.0302 (7)	0.0343 (7)	0.0101 (6)	0.0145 (6)	0.0033 (6)
C13	0.0385 (8)	0.0273 (7)	0.0346 (7)	0.0051 (5)	0.0112 (6)	0.0090 (5)
C14	0.0365 (7)	0.0352 (7)	0.0340 (7)	0.0004 (6)	0.0161 (6)	0.0089 (6)
C15	0.0293 (6)	0.0296 (7)	0.0328 (7)	0.0019 (5)	0.0144 (5)	0.0026 (5)
C16	0.0282 (7)	0.0208 (6)	0.0227 (6)	0.0037 (4)	0.0084 (5)	0.0027 (4)
C17	0.0289 (6)	0.0245 (6)	0.0278 (6)	0.0005 (5)	0.0096 (5)	0.0006 (5)
C18	0.0316 (7)	0.0311 (7)	0.0380 (7)	0.0044 (5)	0.0165 (6)	0.0056 (6)
C19	0.0431 (9)	0.0401 (8)	0.0306 (8)	0.0128 (6)	0.0203 (7)	0.0070 (5)
C20	0.0435 (8)	0.0387 (8)	0.0256 (7)	0.0072 (6)	0.0106 (6)	−0.0044 (6)
C21	0.0319 (7)	0.0287 (7)	0.0285 (7)	0.0004 (5)	0.0079 (5)	−0.0030 (5)

### Geometric parameters (Å, °)

**Table:**

P1—C8	1.8345 (12)	C10—C15	1.4010 (18)
P1—C16	1.8357 (14)	C11—C12	1.3930 (18)
P1—C10	1.8408 (12)	C11—H11	0.9500
N1—C1	1.3196 (17)	C12—C13	1.386 (2)
N1—C9	1.3718 (16)	C12—H12	0.9500
C1—C2	1.410 (2)	C13—C14	1.386 (2)
C1—H1	0.9500	C13—H13	0.9500
C2—C3	1.359 (2)	C14—C15	1.3887 (19)
C2—H2	0.9500	C14—H14	0.9500
C3—C4	1.4167 (19)	C15—H15	0.9500
C3—H3	0.9500	C16—C17	1.3953 (18)
C4—C5	1.417 (2)	C16—C21	1.3982 (18)
C4—C9	1.4200 (17)	C17—C18	1.3869 (19)
C5—C6	1.367 (2)	C17—H17	0.9500
C5—H5	0.9500	C18—C19	1.391 (2)
C6—C7	1.4158 (18)	C18—H18	0.9500
C6—H6	0.9500	C19—C20	1.390 (2)
C7—C8	1.3783 (18)	C19—H19	0.9500
C7—H7	0.9500	C20—C21	1.389 (2)
C8—C9	1.4275 (16)	C20—H20	0.9500
C10—C11	1.3964 (18)	C21—H21	0.9500
			
C8—P1—C16	101.70 (6)	C12—C11—C10	120.61 (12)
C8—P1—C10	102.01 (6)	C12—C11—H11	119.7
C16—P1—C10	101.34 (6)	C10—C11—H11	119.7
C1—N1—C9	117.24 (11)	C13—C12—C11	120.23 (13)
N1—C1—C2	124.12 (13)	C13—C12—H12	119.9
N1—C1—H1	117.9	C11—C12—H12	119.9
C2—C1—H1	117.9	C14—C13—C12	119.79 (13)
C3—C2—C1	119.18 (12)	C14—C13—H13	120.1
C3—C2—H2	120.4	C12—C13—H13	120.1
C1—C2—H2	120.4	C13—C14—C15	120.16 (13)
C2—C3—C4	119.39 (13)	C13—C14—H14	119.9
C2—C3—H3	120.3	C15—C14—H14	119.9
C4—C3—H3	120.3	C14—C15—C10	120.76 (12)
C3—C4—C5	123.26 (12)	C14—C15—H15	119.6
C3—C4—C9	117.40 (12)	C10—C15—H15	119.6
C5—C4—C9	119.33 (12)	C17—C16—C21	118.87 (12)
C6—C5—C4	120.18 (12)	C17—C16—P1	123.58 (10)
C6—C5—H5	119.9	C21—C16—P1	117.55 (10)
C4—C5—H5	119.9	C18—C17—C16	120.56 (13)
C5—C6—C7	120.63 (12)	C18—C17—H17	119.7
C5—C6—H6	119.7	C16—C17—H17	119.7
C7—C6—H6	119.7	C17—C18—C19	120.14 (14)
C8—C7—C6	121.10 (12)	C17—C18—H18	119.9
C8—C7—H7	119.4	C19—C18—H18	119.9
C6—C7—H7	119.4	C20—C19—C18	119.85 (13)
C7—C8—C9	118.93 (11)	C20—C19—H19	120.1
C7—C8—P1	124.99 (9)	C18—C19—H19	120.1
C9—C8—P1	116.00 (9)	C21—C20—C19	119.97 (13)
N1—C9—C4	122.64 (11)	C21—C20—H20	120.0
N1—C9—C8	117.52 (11)	C19—C20—H20	120.0
C4—C9—C8	119.83 (11)	C20—C21—C16	120.61 (13)
C11—C10—C15	118.41 (12)	C20—C21—H21	119.7
C11—C10—P1	124.26 (10)	C16—C21—H21	119.7
C15—C10—P1	117.09 (10)		
			
C9—N1—C1—C2	0.41 (19)	C8—P1—C10—C11	103.60 (11)
N1—C1—C2—C3	0.8 (2)	C16—P1—C10—C11	−1.13 (12)
C1—C2—C3—C4	−1.8 (2)	C8—P1—C10—C15	−82.02 (11)
C2—C3—C4—C5	−178.48 (13)	C16—P1—C10—C15	173.26 (10)
C2—C3—C4—C9	1.73 (19)	C15—C10—C11—C12	−1.01 (19)
C3—C4—C5—C6	179.91 (13)	P1—C10—C11—C12	173.30 (10)
C9—C4—C5—C6	−0.3 (2)	C10—C11—C12—C13	0.8 (2)
C4—C5—C6—C7	0.1 (2)	C11—C12—C13—C14	0.7 (2)
C5—C6—C7—C8	0.3 (2)	C12—C13—C14—C15	−2.0 (2)
C6—C7—C8—C9	−0.50 (19)	C13—C14—C15—C10	1.8 (2)
C6—C7—C8—P1	176.31 (10)	C11—C10—C15—C14	−0.30 (19)
C16—P1—C8—C7	107.19 (11)	P1—C10—C15—C14	−175.03 (11)
C10—P1—C8—C7	2.74 (13)	C8—P1—C16—C17	−20.27 (12)
C16—P1—C8—C9	−75.92 (10)	C10—P1—C16—C17	84.70 (11)
C10—P1—C8—C9	179.64 (9)	C8—P1—C16—C21	159.62 (10)
C1—N1—C9—C4	−0.49 (18)	C10—P1—C16—C21	−95.41 (11)
C1—N1—C9—C8	179.06 (11)	C21—C16—C17—C18	0.00 (19)
C3—C4—C9—N1	−0.56 (18)	P1—C16—C17—C18	179.90 (10)
C5—C4—C9—N1	179.64 (11)	C16—C17—C18—C19	0.0 (2)
C3—C4—C9—C8	179.90 (11)	C17—C18—C19—C20	0.1 (2)
C5—C4—C9—C8	0.09 (18)	C18—C19—C20—C21	−0.2 (2)
C7—C8—C9—N1	−179.27 (11)	C19—C20—C21—C16	0.2 (2)
P1—C8—C9—N1	3.64 (15)	C17—C16—C21—C20	−0.13 (19)
C7—C8—C9—C4	0.30 (18)	P1—C16—C21—C20	179.97 (10)
P1—C8—C9—C4	−176.79 (9)		
